# Supplementary figures and images for: Serrated polyps in patients with ulcerative colitis: Unique clinicopathological and biological characteristics
Source: PLoS One. 2023 Feb 24;18(2):e0282204. doi: 10.1371/journal.pone.0282204 (PMC9955668; doi:10.1371/journal.pone.0282204)

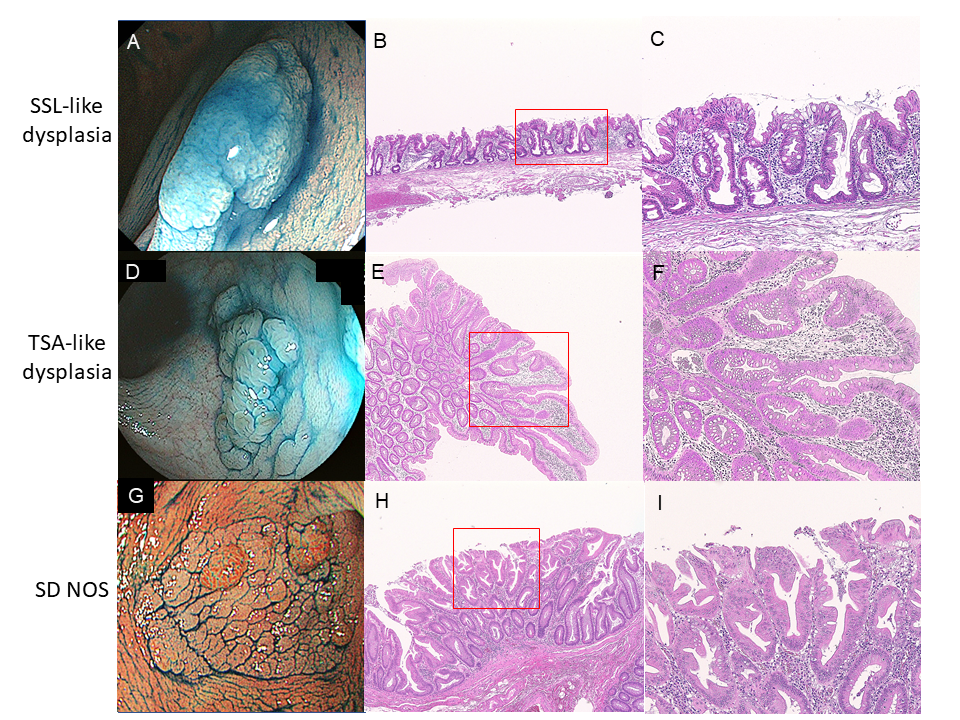

Supplement: S1 Fig — (A–C) Representative case of SSL-like dysplasia. (A) A 12-mm non-polypoid (superficial elevated) lesion was detected in the sigmoid colon. (B, C) Histologically, distorted serrated crypts with dilated L- and inverted T-shaped crypts are visible, resembling SSLs in individuals without IBD. (C) Magnified view of the box outlined in orange in (B). (D–F) Representative case of TSA-like dysplasia. (D) A 15-mm polypoid (sessile) lesion was detected in the sigmoid colon. (E, F) Histologically, a villous pattern with eosinophilic cytoplasm and ectopic crypt formation is visible, resembling TSAs in individuals without IBD. (F) Microscopic view of the box outlined in orange in (E). (G–I) Representative case of SD NOS. (G) A 20-mm non-polypoid (superficial elevated) lesion was detected in the descending colon. (H, I) Histologically, the lesion has a serrated structure resembling a TSA, but does not show a villous or papillary structure or ectopic crypt formation, which that would have led to a definitive diagnosis of TSA. (I) Microscopic view of box outlined in orange in (H). IBD, inflammatory bowel disease; SD NOS, serrated dysplasia not otherwise specified; SSL, sessile serrated lesion; TSA, traditional serrated adenoma. (TIF) [file pone.0282204.s001.tif]
