# Supplementary material for: Serrated polyps in patients with ulcerative colitis: Unique clinicopathological and biological characteristics
Source: PLoS One. 2023 Feb 24;18(2):e0282204. doi: 10.1371/journal.pone.0282204 (PMC9955668; doi:10.1371/journal.pone.0282204)
Supplement: S1 Table — (DOCX) [file pone.0282204.s002.docx]

**S1 Table. Comparison of clinical and endoscopic characteristics between SSL/SSL-dysplasias in patients with UC and SSLs in patients without IBD**

|  | SSL or SSL-like dysplasias in patients with UC (n = 24) | SSLs in patients without IBD  (n =219) |
| --- | --- | --- |
| Neoplasia location |  |  |
| Proximal colon | 19 (79) | 182 (83) |
| Distal colon | 5 (21) | 37 (17) |
| Size, mm ^a^ | 9 (7–17) | 16 (10–30) |
| Morphology |  |  |
| Polypoid | 1 (4) | 17 (8) |
| Non-polypoid | 23 (96) | 202 (92) |
| Distinct border | 24 (100) | 219 (100) |
| Histopathology ^a^ |  |  |
| SSL | 9 (37) | 219 (100) |
| SSL-like dysplasia | 15 (63) | 0 (0) |

IBD, inflammatory bowel disease; SSL, sessile serrated lesion; UC, ulcerative colitis

^a^ *P* < 0.05 between SSL/SSL-dysplasias in patients with UC and SSLs in patients without IBD
