# Supplementary material for: Serrated polyps in patients with ulcerative colitis: Unique clinicopathological and biological characteristics
Source: PLoS One. 2023 Feb 24;18(2):e0282204. doi: 10.1371/journal.pone.0282204 (PMC9955668; doi:10.1371/journal.pone.0282204)
Supplement: S2 Table — (DOCX) [file pone.0282204.s003.docx]

**S2 Table. Comparison of clinical and endoscopic characteristics between TSA/TSA-dysplasias in patients with UC and TSAs in patients without IBD**

|  | TSA or TSA-like dysplasias in patients with UC (n = 7) | TSAs in patients without IBDs (n = 30) |
| --- | --- | --- |
| Neoplasia location |  |  |
| Proximal colon | 1 (14) | 6 (20) |
| Distal colon | 6 (86) | 24 (80) |
| Size, mm ^a^ | 5 (5–12) | 17 (10–24) |
| Morphology |  |  |
| Polypoid | 5 (71) | 18 (60) |
| Non-polypoid | 2 (29) | 12 (40) |
| Distinct border | 7 (100) | 30 (100) |
| Histopathology ^a^ |  |  |
| TSA | 1 (14) | 30 (100) |
| TSA-like dysplasia | 6 (86) | 0 (0) |

IBD, inflammatory bowel disease; TSA, traditional serrated adenoma; UC, ulcerative colitis

^a^ *P* < 0.05 between TSA/TSA-dysplasias in patients with UC and TSAs in patients without IBD
