# Supplementary material for: Serrated polyps in patients with ulcerative colitis: Unique clinicopathological and biological characteristics
Source: PLoS One. 2023 Feb 24;18(2):e0282204. doi: 10.1371/journal.pone.0282204 (PMC9955668; doi:10.1371/journal.pone.0282204)
Supplement: S3 Table — (DOCX) [file pone.0282204.s004.docx]

**S3 Table. Comparison of the Clinical Characteristics Among the Neoplasia Categories**

|  | In colitis-affected segments | | In colitis-unaffected segments | |
| --- | --- | --- | --- | --- |
|  | Serrated polyps in colitis-affected segments  (n = 26) | Conventional dysplasia  (n = 132) | Serrated polyps in colitis-unaffected segments  (n = 10) | Sporadic adenomas  (n = 51) |
| Men^a,b,d,f^ | 23 (88) | 85 (65) | 2 (20) | 36 (71) |
| Age at diagnosis of neoplasia, years^c,e^ | 50 (39–62) | 57 (44–69) | 44 (38–78) | 67 (56–73) |
| Age at diagnosis of UC, years^c,e^ | 37 (22–43) | 40 (28–57) | 39 (34–58) | 52 (42–59) |
| Duration of UC at diagnosis of neoplasia, years^b^ | 12.1 (7.8–20.4) | 10.0 (4.5–20.9) | 5.5 (1.2–17.1) | 11.1 (5.2–22.1) |
| Disease type |  |  |  |  |
| Extensive colitis (E3)^b,c,d,e,f^ | 23 (88) | 98 (74) | 4 (40) | 5 (10) |
| Left-sided colitis (E2)^a,c,e,f^ | 2 (8) | 32 (24) | 2 (20) | 29 (57) |
| Proctitis (E1)^b,c,d,e^ | 1 (4) | 2 (2) | 4 (40) | 17 (33) |
| History of persistent active colitis^c,e^ | 15 (58) | 69 (52) | 2 (20) | 11 (22) |
| History of severe disease of UC^e^ | 6 (23) | 31 (24) ^e^ | 0 (0) | 3 (7) |
| Previous UC treatment, n (%) |  |  |  |  |
| 5-ASA/SASP | 25 (96) | 128 (97) | 8 (80) | 48 (94) |
| Corticosteroid^e^ | 17 (62) | 83 (63) | 4 (40) | 22 (43) |
| Immunomodulater^a,c,^ | 12 (47) | 31 (23) | 2 (20) | 6 (12) |
| Anti-TNF antibody^c,e^ | 6 (23) | 14 (11) | 1 (10) | 0 (0) |
| Calcineurin inhibitors | 3 (12) | 10 (8) | 0 (0) | 1 (2) |
| JAK inhibitor | 0 (0) | 0 (0) | 0 (0) | 0 (0) |
| α4β7 inhibitor | 0 (0) | 0 (0) | 0 (0) | 0 (0) |
| IL12/23 inhibitor | 0 (0) | 0 (0) | 0 (0) | 0 (0) |
| Neoplasia location^b,d,e^ |  |  |  |  |
| Proximal colon | 11 (42) | 35 (27) | 10 (100) | 38 (75) |
| Distal colon | 15 (58) | 97 (73) | 0 (0) | 13 (25) |
| Size, mm^c,e,f^ | 9 (7–19) | 9 (6–18) | 10 (8–19) | 4 (3–5) |
| Morphology^a,c,e,f^ |  |  |  |  |
| Polypoid | 6 (23) | 71 (54) | 2 (20) | 40 (78) |
| Non-polypoid | 20 (77) | 61 (46) | 8 (80) | 11 (22) |
| Treatment of neoplasia |  |  |  |  |
| ER^b^ | 22 (85) | 84 (64) | 8 (80) | 39 (76) |
| Surgery^a,e^ | 1 (4) | 37 (28) | 0 (0) | 0 (0) |
| Not resected^e^ | 3 (11) | 11 (8) | 2 (0) | 12 (24) |

Data are presented as n (%) or median (interquartile range).

ER, endoscopic resection; IL, interleukin; JAK, Janus kinase; SASP, salazosulfapyridine; TNF, tumor necrosis factor; UC, ulcerative colitis, 5-ASA, 5-aminosalicylic acid

^a^*P* < 0.05 between serrated polyps in colitis-affected segments and conventional dysplasia

^b^*P* < 0.05 between serrated polyps in colitis-affected segments and serrated polyps in colitis-unaffected segments

^c^*P* < 0.05 between serrated polyps in colitis-affected segments and adenomas

^d^*P* < 0.05 between conventional dysplasia and serrated polyps in colitis-unaffected segments

^e^*P* < 0.05 between conventional dysplasia and adenomas

^f^*P* < 0.05 between serrated polyps in colitis-unaffected segments and adenomas
