# Supplementary material for: Serrated polyps in patients with ulcerative colitis: Unique clinicopathological and biological characteristics
Source: PLoS One. 2023 Feb 24;18(2):e0282204. doi: 10.1371/journal.pone.0282204 (PMC9955668; doi:10.1371/journal.pone.0282204)
Supplement: S4 Table — (DOCX) [file pone.0282204.s005.docx]

**S4 Table. Genetic and epigenetic analyses of serrated polyps and colitis-associated cancer**

|  | serrated polyps in colitis-affected segments  (n = 13) | invasive cancers in colitis-affected segments  (n = 16) |
| --- | --- | --- |
| *KRAS* | 8 (62) | 2 (13) |
| *BRAF* | 4 (31) | 0 (0) |
| CIMP-positive | 5 (38) | 3 (19) |

CIMP, CpG island methylation phenotype
