# Supplementary material for: Serrated polyps in patients with ulcerative colitis: Unique clinicopathological and biological characteristics
Source: PLoS One. 2023 Feb 24;18(2):e0282204. doi: 10.1371/journal.pone.0282204 (PMC9955668; doi:10.1371/journal.pone.0282204)
Supplement: S1 File — (DOCX) [file pone.0282204.s006.docx]

**S1 File. The details of the laser microdissection system, DNA extraction, and genetic and epigenetic analyses.**

Isolation of Neoplastic Lesions by Laser-Capture Microdissection and DNA Extraction

Laser-capture microdissection (LMD) was performed to isolate neoplasia from discrete background neoplasia or mucosae. Appropriate tissue blocks were selected for DNA extraction from the resected neoplasms. A Leica LMD 6500 Laser Microdissection System (Leica Microsystems, Wetzlar, Germany) was used to isolate neoplasms from formalin-fixed, paraffin-embedded (FFPE) tissue sections. The neoplasms were selectively dissected by microscopic examination.

DNA was extracted from isolated tissues using the Promega ReliaPrep FFPE gDNA Miniprep System (Promega, Fitchburg, WI) for FFPE samples and the Promega ReliaPrep gDNA Tissue Miniprep System for frozen samples according to the manufacturer’s protocols. Nucleic acid concentrations were measured using the Qubit dsDNA HS kit and Qubit Fluorometer (Thermo Fisher Scientific, Waltham, MA).

Analysis of KRAS and BRAF Mutations

To evaluate the frequency of *KRAS* and *BRAF* mutations, serrated polyps and invasive cancers with adequate DNA yields from patients with ulcerative colitis were analyzed using the QX200 droplet digital polymerase chain reaction (PCR) system (Bio-Rad Laboratories, Hercules, CA) according to the manufacturer’s protocol. The PrimePCR ddPCR Mutation Detection Assay Kit was used to detect *KRAS* mutations (G12D, G12V, and G12R) and *BRAF* mutation (V600E) (Bio-Rad Laboratories). If samples did not show *KRAS* G12D, G12V, and G12R mutations, the LBx Probe *KRAS* G12/13 Screen (G12A, G12C, G12D, G12F, G12G, G12L, G12S, G12V, G13A, G13C, G13D, G13G, G13R, G13S, and G13V; RIKEN GENESIS, Tokyo, Japan) was used. We recorded variants at ≥2% allelic frequency and ≥3× mutant allele coverage.

Analysis of CpG Island Hypermethylation Phenotype (CIMP) Status

To assess the CIMP status, serrated polyps and invasive cancers with adequate DNA yields from patients with ulcerative colitis were analyzed using a five-gene signature as follows: *CDKN2A*, *MINT1*, *MINT2*, *MINT31*, and *MLH1*. [1, 2] Gene body methylation was quantified using MethyLight (Qiagen, Venlo, the Netherlands), a methylation-specific probe-based real-time PCR method, according to the manufacturer’s protocol. [1, 2] Gene body methylation of target genes was normalized to that of *Alu* (internal control). DNA methylation was expressed as the percentage of methylated reference (PMR) = 100 × ([methylated reaction/*Alu*] sample/[methylated reaction/*Alu*] M.SssI-reference). [2] Each marker was considered methylated when PMR ≥ 4. [2] According to the established criteria, [1, 2] samples were defined as CIMP-positive (CIMP+) if at least three of the five aforementioned genes were methylated and CIMP-negative (CIMP−) if two or fewer genes were methylated.

**References**

1. Toyota M, Ahuja N, Ohe-Toyota M, Herman JG, Baylin SB, Issa JP. CpG island methylator phenotype in colorectal cancer. Proc Natl Acad Sci U S A. 1999;96:8681-6.

2. Weisenberger DJ, Siegmund KD, Campan M, Young J, Long TI, Faasse MA, et al. CpG island methylator phenotype underlies sporadic microsatellite instability and is tightly associated with BRAF mutation in colorectal cancer. Nat Genet. 2006;38:787-93.
