# Supplementary material for: Serrated polyps in patients with ulcerative colitis: Unique clinicopathological and biological characteristics
Source: PLoS One. 2023 Feb 24;18(2):e0282204. doi: 10.1371/journal.pone.0282204 (PMC9955668; doi:10.1371/journal.pone.0282204)
Supplement: S1 Checklist — (DOCX) [file pone.0282204.s007.docx]

STROBE Statement—checklist of items that should be included in reports of observational studies

|  | Item No. | Recommendation | Page  No. | Relevant text from manuscript |
| --- | --- | --- | --- | --- |
| **Title and abstract** | 1 | (*a*) Indicate the study’s design with a commonly used term in the title or the abstract | 3 | a retrospective cohort study |
|  |  | (*b*) Provide in the abstract an informative and balanced summary of what was done and what was found | 3,4 | Methods and Results sections of abstract |
| Introduction | | | |  |
| Background/rationale | 2 | Explain the scientific background and rationale for the investigation being reported | 5,6 | First, second, and third paragraphs of Introduction |
| Objectives | 3 | State specific objectives, including any prespecified hypotheses | 6 | Third paragraph of Introduction |
| Methods | | | |  |
| Study design | 4 | Present key elements of study design early in the paper | 6–8 | Data Collection, Patients, and Neoplasia and Classification and Histological Evaluation of Neoplasia subsection |
| Setting | 5 | Describe the setting, locations, and relevant dates, including periods of recruitment, exposure, follow-up, and data collection | 6-8 | Data Collection, Patients, and Neoplasia subsection |
| Participants | 6 | (*a*) *Cohort study*—Give the eligibility criteria, and the sources and methods of selection of participants. Describe methods of follow-up  *Case-control study*—Give the eligibility criteria, and the sources and methods of case ascertainment and control selection. Give the rationale for the choice of cases and controls  *Cross-sectional study*—Give the eligibility criteria, and the sources and methods of selection of participants | 6-8 | Data Collection, Patients, and Neoplasia subsection |
|  |  | (*b*) *Cohort study*—For matched studies, give matching criteria and number of exposed and unexposed  *Case-control study*—For matched studies, give matching criteria and the number of controls per case | Not applicable | Not applicable |
| Variables | 7 | Clearly define all outcomes, exposures, predictors, potential confounders, and effect modifiers. Give diagnostic criteria, if applicable | 7,8 | Classification and Histological Evaluation of Neoplasia subsection |
| Data sources/ measurement | 8* | For each variable of interest, give sources of data and details of methods of assessment (measurement). Describe comparability of assessment methods if there is more than one group | 6, 7, 9 | Data Collection, Patients, and Neoplasia subsection and DNA Extraction and Genetic and Epigenetic Analyses subsection |
| Bias | 9 | Describe any efforts to address potential sources of bias | 6,7 | First paragraph of Data Collection, Patients, and Neoplasia subsection and first paragraph of Classification and Histological Evaluation of Neoplasia subsection |
| Study size | 10 | Explain how the study size was arrived at | 6,7 | Sample size was determined after application of eligibility criteria to all consecutive UC patients who underwent total colonoscopy at the author’s center between 2000 and 2020 |

Continued on next page

| Quantitative variables | 11 | Explain how quantitative variables were handled in the analyses. If applicable, describe which groupings were chosen and why | 7, 9 | Second paragraph of Data Collection, Patients, and Neoplasia subsection and statistical analysis subsection |
| --- | --- | --- | --- | --- |
| Statistical methods | 12 | (*a*) Describe all statistical methods, including those used to control for confounding | 9 | Statistical analysis subsection |
|  |  | (*b*) Describe any methods used to examine subgroups and interactions | 9 | Statistical analysis subsection |
|  |  | (*c*) Explain how missing data were addressed | Not applicable | Not applicable |
|  |  | (*d*) *Cohort study*—If applicable, explain how loss to follow-up was addressed  *Case-control study*—If applicable, explain how matching of cases and controls was addressed  *Cross-sectional study*—If applicable, describe analytical methods taking account of sampling strategy | Not applicable | Not applicable |
|  |  | (*e*) Describe any sensitivity analyses | Not applicable | Not applicable |
| Results | | | | |
| Participants | 13* | (a) Report numbers of individuals at each stage of study—eg numbers potentially eligible, examined for eligibility, confirmed eligible, included in the study, completing follow-up, and analysed | 10-17 | Prevalence of Serrated Polyps in Patients with UC subsection and Clinical Characteristics of Serrated Polyps in Colitis-Affected Segments subsection. Second paragraph of Locational and Biological Characteristics of Serrated Polyps in Patients with UC subsection |
|  |  | (b) Give reasons for non-participation at each stage | Not applicable | Not applicable |
|  |  | (c) Consider use of a flow diagram | 10,11 | First paragraph of Prevalence of Serrated Polyps in Patients with UC subsection (Figure 1) |
| Descriptive data | 14* | (a) Give characteristics of study participants (eg demographic, clinical, social) and information on exposures and potential confounders | 11-16 | Clinical Characteristics of Patients with Serrated Polyps and Other Neoplasms subsection and Table 1. Clinical Characteristics of Serrated Polyps in Colitis-Affected Segments subsection and Table 2. |
|  |  | (b) Indicate number of participants with missing data for each variable of interest | 17 | Second paragraph and Locational and Biological Characteristics of Serrated Polyps in Patients with UC subsection. |
|  |  | (c) *Cohort study*—Summarise follow-up time (eg, average and total amount) | Not applicable | Not applicable |
| Outcome data | 15* | *Cohort study*—Report numbers of outcome events or summary measures over time | Not applicable | Not applicable |
|  |  | *Case-control study—*Report numbers in each exposure category, or summary measures of exposure |  |  |
|  |  | *Cross-sectional study—*Report numbers of outcome events or summary measures |  |  |
| Main results | 16 | (*a*) Give unadjusted estimates and, if applicable, confounder-adjusted estimates and their precision (eg, 95% confidence interval). Make clear which confounders were adjusted for and why they were included | Not applicable | Not applicable |
|  |  | (*b*) Report category boundaries when continuous variables were categorized | Not applicable | Not applicable |
|  |  | (*c*) If relevant, consider translating estimates of relative risk into absolute risk for a meaningful time period | Not applicable | Not applicable |

Continued on next page

| Other analyses | 17 | Report other analyses done—eg analyses of subgroups and interactions, and sensitivity analyses | Not applicable | Not applicable |
| --- | --- | --- | --- | --- |
| Discussion | | | | |
| Key results | 18 | Summarise key results with reference to study objectives | 18 | First paragraph of Discussion |
| Limitations | 19 | Discuss limitations of the study, taking into account sources of potential bias or imprecision. Discuss both direction and magnitude of any potential bias | 22 | Seventh paragraph of Discussion |
| Interpretation | 20 | Give a cautious overall interpretation of results considering objectives, limitations, multiplicity of analyses, results from similar studies, and other relevant evidence | 22,23 | Conclusion secession |
| Generalisability | 21 | Discuss the generalisability (external validity) of the study results | 18-22 | Second to Sixth paragraphs of Discussion |
| Other information | |  | | |
| Funding | 22 | Give the source of funding and the role of the funders for the present study and, if applicable, for the original study on which the present article is based | Described in submission web site | We described that we had no financial support and potential competing interest in the study. |

*Give information separately for cases and controls in case-control studies and, if applicable, for exposed and unexposed groups in cohort and cross-sectional studies.

**Note:** An Explanation and Elaboration article discusses each checklist item and gives methodological background and published examples of transparent reporting. The STROBE checklist is best used in conjunction with this article (freely available on the Web sites of PLoS Medicine at http://www.plosmedicine.org/, Annals of Internal Medicine at http://www.annals.org/, and Epidemiology at http://www.epidem.com/). Information on the STROBE Initiative is available at www.strobe-statement.org.
